# Examining Stroke Symptom Messages Implemented Globally: A Need for Contextually Relevant Stroke Symptom Messaging

**DOI:** 10.1111/jnu.70059

**Published:** 2025-12-17

**Authors:** Hardeep Singh, Sarah Belson, Jennifer E. S. Beauchamp, Michelle L. A. Nelson

**Affiliations:** ^1^ Department of Occupational Science and Occupational Therapy, Temerty Faculty of Medicine University of Toronto Toronto Ontario Canada; ^2^ Rehabilitation Sciences Institute, Temerty Faculty of Medicine University of Toronto Toronto Ontario Canada; ^3^ World Stroke Organization Geneva Switzerland; ^4^ Stroke Association London UK; ^5^ Department of Research Cizik School of Nursing at UTHealth Houston Houston Texas USA; ^6^ UTHealth Houston Institute for Stroke and Cerebrovascular Diseases Houston Texas USA; ^7^ Lunenfeld‐Tanenbaum Research Institute, Sinai Health Toronto Ontario Canada; ^8^ Institute of Health Policy, Management and Evaluation, Dalla Lana School of Public Health University of Toronto Toronto Ontario Canada

**Keywords:** stroke awareness, stroke response, survey

## Abstract

**Background:**

Stroke is a global health concern. A timely response to a stroke can help reduce morbidity and mortality. However, barriers to timely response include poor recognition of stroke symptoms. Stroke symptom messages are designed to increase stroke recognition and encourage individuals to seek urgent medical assistance. The Face, Arm, Speech, Time (FAST) and Balance, Eyes, Face, Arm, Speech, Time (BE FAST) are commonly used stroke symptom messages shown to improve stroke symptom recognition and response. However, cultural factors and language differences may limit the effectiveness of stroke symptom messages and their acceptability in different countries and contexts. There has not been a comprehensive examination of the stroke symptom messages used worldwide and how these messages have been adapted in various settings.

**Aims:**

We explored what stroke response messages are being used globally, and the contextual factors that influence the adoption of a stroke response mnemonic in different settings.

**Methods:**

A 14‐item survey was disseminated by the World Stroke Organization to its networks. The survey contained open‐ and closed‐ended questions and allowed uploading relevant stroke symptom campaign materials. The survey was analyzed using descriptive statistics and a content analysis.

**Results:**

All except one survey respondent used a stroke symptom message. Fifteen respondents (27%) reported they did not translate their stroke awareness messaging. Of these 15 respondents, they used the English versions of FAST (*n* = 8), BE FAST (*n* = 4), and both FAST and BE FAST (*n* = 3). Forty respondents (71%) reported that they/their organization used an acronym to raise public awareness of the signs/symptoms of stroke that was different from FAST or BE FAST (English), many of which were direct or indirect translations or influenced by FAST and BE FAST. Survey responses shared insights and recommendations related to the content, tailoring and dissemination of stroke symptom messages.

**Conclusions:**

Study findings highlight the global use of stroke symptom messages and their contextual adaptations to fit diverse settings and contexts. The challenges in applying universal or commonly used stroke symptom messages to different contexts were highlighted.

**Clinical Relevance:**

Nurses could have a key role in raising awareness of stroke symptoms and the development of locally adapted stroke symptom messages.

## 1 Background

Stroke is a global health concern. As a common cause of death and disability globally, stroke is a public health concern among non‐communicable diseases (Feigin et al. 2024; Feigin et al. 2023). Globally, the incidence of stroke has increased between 1990 and 2021, impacting health systems across the world (Feigin et al. 2024; Feigin et al. 2023). Stroke‐related mortality is also increasing, and may double between 2020 and 2050 (Feigin et al. 2023). Additionally, disparities are found across countries in the burden of stroke and stroke‐related risk factors, with low‐ and middle‐income countries having a disproportionately higher burden compared to high‐income countries (Feigin et al. 2023, 2024; Yu et al. 2025).

There remains a global challenge related to poor awareness and understanding of stroke (Waweru et al. 2024). Researchers have noted the need for greater public awareness about the signs of stroke and the importance of timely emergency response (Feigin et al. 2023). Prompt recognition of stroke signs and immediate health treatment can reduce the risk of mortality and long‐term disability after a stroke (Goyal et al. 2015; Mellon et al. 2015). However, limited awareness and understanding of stroke symptoms and the importance of timely medical intervention can delay access to critical acute treatments (Hickey et al. 2018), such as thrombolysis and thrombectomy (Wardlaw et al. 2014). Researchers have indicated that stroke outcomes could be improved through ongoing stroke symptom awareness campaigns that can improve public awareness of stroke signs and minimize delays in seeking immediate medical care (Feigin et al. 2023, 2024). Additionally, contextually relevant education and messaging may be needed to address stroke literacy disparities (Zhao et al. 2018; Zhao and Liu 2016).

The Face, Arm, Speech, Time (FAST) and Balance, Eyes, Face, Arm, Speech, Time (BE FAST) are two widely known campaigns globally aimed at increasing stroke symptom awareness and promoting rapid response to the recognition of signs of stroke among healthcare professionals and the public (Harbison et al. 2003; Hogge et al. 2024). FAST was developed to help ambulance personnel quickly recognize and respond to stroke symptoms, addressing the issue of stroke misdiagnosis (Harbison et al. 2003). BE FAST builds upon FAST by including additional stroke signs (Aroor et al. 2017). These stroke symptom awareness campaigns have been adopted in both English‐speaking countries (Chen et al. 2021) and those where the general population less commonly speaks English (Kim et al. 2011; Zhao and Liu 2016).

FAST and BE FAST campaigns have demonstrated some positive impacts on stroke symptom recognition (Kleindorfer et al. 2008; Pierce et al. 2011; Wall et al. 2008). Studies have also noted improvements in responses to stroke, including accessing emergency medical services for suspected stroke and reduced delays in seeking acute medical treatment (Bray et al. 2011; Flynn et al. 2014; Wolters et al. 2015). However, critiques of the FAST and BE FAST note that their development and primary use have been limited to English‐speaking countries (Zhao et al. 2018; Zhao and Liu 2016). Given that only about 5% of the global population speaks English as a primary language, there may be a critical gap in effective and culturally appropriate stroke symptom messages for non‐English‐speaking populations (Zhao et al. 2018). To reduce language barriers and widen their utility (Waweru et al. 2024), the FAST and BE FAST acronyms have been translated into multiple languages, including Swahili (Waweru et al. 2024), Spanish (Banerjee et al. 2022; Castro et al. 2022), and Swedish (Nordanstig et al. 2017). However, there are concerns that such language translations may not fully convey the original messages and could reduce memorability/recall among non‐English‐speaking populations (Beauchamp et al. 2022; Zhao et al. 2018).

Some medical professionals have expressed a preference for different stroke awareness interventions due to language barriers associated with FAST and BE FAST (Zhao et al. 2018). For example, Stroke 112 was developed to eliminate language barriers and demonstrated greater acceptance than FAST among neurologists in China (Zhao et al. 2018). Even within English‐speaking countries, preferences for FAST and BE FAST may vary (Hogge et al. 2024), and public awareness of these acronyms, particularly among racialized populations, remains low (Bietzk et al. 2012). To date, there has been no comprehensive examination of the stroke symptom awareness messages used worldwide or how these messages have been contextually adapted to different settings.

### 1.1 Aims

To explore what stroke response messages are used globally and the contextual factors influencing their adoption in different settings.

## 2 Methods

### 2.1 Design

This study employed a cross‐sectional, descriptive survey design to explore stroke symptom messaging used across different countries and its contextual adaptations.

### 2.2 Ethical Considerations

Ethics approval was granted from the University of Toronto for the analysis of data previously collected as part of an organizational program improvement initiative. Participants received information about the study's purpose, confidentiality, use of the data, and the voluntary nature of their participation in the survey. No personal information was collected. De‐identified data are securely managed with password protection on an institutional server and will be destroyed 5 years after study completion, as per institutional guidance.

### 2.3 Population

Stroke organizations worldwide.

### 2.4 Survey Sample and Sampling

We used multiple sampling strategies to recruit our sample, including convenience, purposeful and snowball sampling methods (Palinkas et al. 2015). The organizations invited to participate were members of two umbrella not‐for‐profit stroke organizations that convene and support local stroke organizations. One organization represents members from across the world, and the other has members from Europe specifically. These organizations sent their members an email invitation to participate with introductory information (e.g., purpose, survey instructions, and confidentiality). The first organization had membership from stroke support organizations (SSOs) and professional societies (*n* = 105). The second organization had members from SSOs (*n* = 38). These members were selected due to their accessibility to the team and knowledge and experience with stroke response messaging. Additionally, some organizations shared the invitation with their contacts.

### 2.5 Data Collection

A checklist was used to ensure comprehensive reporting of survey methods (Kelley et al. 2003). A survey enabled data collection from a global sample and generated descriptive data to address our research aims. It was developed by four experts with specialized experience in stroke to ensure appropriateness for an international audience and optimize clarity. Before data collection, the survey was shared with colleagues from stroke organizations for their feedback on the survey questions. The 14‐item voluntary survey was distributed via email invitation. It was available in English, Spanish and French via SurveyMonkey. The survey was open from September 2023 to January 2024, and included yes/no, multiple‐choice, open‐ended questions, and the option to upload files relevant to local stroke symptom messaging (see Appendix A). Survey questions explored which stroke symptom messages were used in different countries and how the messages were contextually adapted. A follow‐up email was sent to three organizations informing them that the survey was now available in the additional languages they had requested (French and Spanish). In December 2023, a reminder email was sent by the second organization due to the low response rate from the region they represented.

### 2.6 Data Analysis

Survey responses were transferred to Microsoft Excel for data cleaning and analysis. The initial clean of the data commenced with creating a backup of the original data. The copied document was formatted, which included numbering each question and wrapping text, capitalizing country names, and converting to English for consistency. During data cleaning, a small number of inconsistent responses were adjusted (i.e., information added (*n* = 3 items) or deleted (*n* = 9)) to reflect explicit information provided elsewhere in the survey from the same respondent. For example, a survey respondent wrote “we use same acronym as FAST” to the question: “If you/your organization use a different acronym than FAST/BE FAST, who was involved in developing this? (Please select all that apply).” This response was deleted because it did not answer the question, and they later indicated that they did not develop an acronym. Another respondent had a missing response for question 4 “If the acronym you/your organization uses to raise public awareness of the signs/symptoms of stroke is different from FAST/BE FAST, have you:” however in their response to question 7 “Please upload what you use to promote stroke signs/symptoms awareness outside of English versions of FAST/BE FAST,” they uploaded a document that indicated the use of an acronym by their organization that was different from FAST/BE FAST. Information regarding their use of the acronym RAPIDO was noted in question 4. The remaining missing values are reported in the results. For example, we note, among the 56 respondents who completed the survey, 32 respondents answered the question: “If you/your organization uses a different acronym than FAST/BE FAST, who was involved in developing this? Please select all that apply.” There were no duplicate survey entries by participants. Outliers were included in the results.

We used Microsoft Excel due to the small dataset size and the limited analysis, which focused on summary statistics and frequencies. Due to our sampling strategy (i.e., use of snowball and purposeful sampling strategies), we could not track the total number of individuals reached; therefore, a traditional response rate could not be determined, which is also acknowledged as a study limitation. Quantitative data were analyzed using descriptive statistics, such as percentages, means, medians and ranges, where applicable. Text responses were categorized to create textual summaries and presented as counts and percentages. A conventional content analysis (Hsieh and Shannon 2005) was used to inductively categorize open‐ended responses to question 14: “Please share any lessons/recommendations about stroke symptom awareness that could be shared with other stroke organizations.” This process involved HS reviewing the qualitative data to gain data immersion and then creating codes, which were short summaries using exact keywords from each participant's response. After coding each response, broader categories were created to group ideas from similar codes. These categories were validated by the team.

## 3 Results

### 3.1 Respondent Characteristics

Fifty‐six surveys were received from 46 countries between 2023 and 2024, including Australia, Barbados, Bhutan, Brazil, Brunei, Bulgaria, Cameroon, Canada (*n* = 2), China, Colombia, Cyprus, Czech Republic, Denmark, Finland, Ghana, India (*n* = 2), Indonesia (*n* = 3), Italy, Japan, Malaysia (*n* = 2), Monaco, Myanmar, Nepal, Netherlands, New Zealand, Nigeria, North Macedonia, Pakistan, Philippines (*n* = 2), Portugal (*n* = 2), South Korea, Rwanda, Serbia, Singapore, Slovakia, Slovenia, South Africa, Spain, Sri Lanka, Sweden, Thailand, Türkiye (*n* = 2), Uganda, Ukraine, and United States (*n* = 3). One respondent did not indicate their country. Participants were asked to select all professional roles that apply to them and included: SSO members (*n* = 42, 75%), health professionals (*n* = 21, 38%), academics/researchers (*n* = 9, 16%), and government/civil servants (*n* = 4, 7%).

### 3.2 Stroke Symptom Messaging Used

All but one respondent used a stroke symptom awareness message. Fifteen respondents (27%) did not indicate translating the stroke awareness messaging, with eight using FAST, four using BE FAST and three using both FAST and BE FAST. Forty respondents (71%) reported that they/their organization used an acronym to raise public awareness of the signs/symptoms of stroke that was different from FAST or BE FAST (English version). Of these 40 respondents, 22 indicated that FAST informed their stroke response messages, 16 respondents indicated that BE FAST informed their stroke response messages, while two respondents did not indicate that their stroke response messages were related to either of these. Table 1 provides examples of the variety of stroke response messages used by respondents/their organizations to raise public awareness of stroke signs and symptoms in their local context (see Table 1). Respondents indicated that language translations addressed language barriers that reduced the effectiveness of the stroke response messages in their local contexts and improved public recall. For instance, in India, musical notes were used to represent stroke symptoms and increase public recall. Additional adaptations included incorporating local emergency numbers.

**TABLE 1:** Examples of stroke response mnemonics reported by survey respondents.

FAST reported by a respondent from New Zealand
BE FAST reported by a respondent from United States
PeRMATA reported by a respondent from Malaysia
Sa Aa Ja Ba Ha Sa reported by a respondent from Nepal
PANTAS reported by a respondent from Brunei
Time is brain reported by a respondent from Portugal
Symptoms of stroke: 3 F's—Face, Fala (=Speech), Força (=Strength) reported by a respondent from Portugal
SA RE GA MA PA DA NE SA reported by a respondent from India
SEGERA KE RS reported by a respondent from Indonesia
Riu, Aixeca els braços, Parla, Ictus? De pressa truca al 112 reported by a respondent from Spain
HITNO: “Urgent” “It is an acronym for: H—Hod ti se promeni—(your) Walking is changed I—Iskrive ti se lice i usta (your) face and mouth are distorted (twisted) T—Teško pomeraš ruku—(you) can hardly move arm N—Noga ti oslabi—(your) leg gets weak O—Otežano govoriš—(you) speak with difficulty Do not wait—call 194” reported by a respondent from Serbia
GROM reported by a respondent from Slovenia
SEGERA KE RS reported by a respondent from Indonesia
VITE reported by a respondent from Monaco
RAPIDO reported by a respondent from Portugal
БРЗО reported by a respondent from North Macedonia
Mond, spraak, arm? Beroerte‐alarm, bel 112! reported by a respondent from the Netherlands
AKUT reported by a respondent from Sweden
SEGERA reported by a respondent from Indonesia
CORRE+ reported by a respondent from Colombia
SAMU‐sorria/abrace/música/urgente (smile, hold, music, urgent) reported by a respondent from Brazil
이웃‐손‐발‐시선 reported by a respondent from South Korea
Think FAST, act FAST. No delays when stroke strikes, one must act FAST‐Ghana
TVÁR RUKA REČ POMOC 155 reported by a respondent from Slovakia
Yüzde kayma, konuşma bozukluğu, kolda güç kaybi. Vakit kaybetmeden 112'yi arayin! reported by a respondent from Türkiye
Stræk Snak Smil reported by a respondent from Denmark

### 3.3 The Need for Stroke Symptom Messages

Respondents highlighted several reasons necessitating efforts to enhance awareness about the signs and symptoms of stroke and the need to seek medical treatment immediately. Twenty‐one respondents (41%) indicated delayed presentations at their regional or local hospitals for medical treatment following a stroke. Twenty‐four respondents (47%) reported low public awareness of stroke signs and symptoms, including a failure to recognize stroke as a medical emergency. Four respondents (8%) reported delayed initiation of stroke protocols and triage by healthcare providers. Three respondents (5%) reported a lack of public knowledge about the appropriate emergency numbers to call or the appropriate medical center to visit, especially in countries with multiple emergency numbers and centers.

### 3.4 Development of Stroke Symptom Messages

Among the 56 respondents who completed the survey, 32 respondents answered the question: “If you/your organization uses a different acronym than FAST/BE FAST, who was involved in developing this? Please select all that apply.” Five respondents indicated that they “have not developed an acronym” and one respondent indicated that they were unsure how it was developed. Table 2 provides an overview of the groups involved in the development of respondents' stroke response messages. Of the remaining 26 respondents, they reported collaborations with health care professionals (*n* = 16, 62%), SSOs (*n* = 15, 58%), academics/researchers (*n* = 13, 50%) were most common. Seventeen of the 26 respondents indicated multiple groups created their stroke symptom messaging. Collaboration between academics/researchers and health care professionals was the most common (*n* = 12), while a few respondents indicated collaboration between their health ministry and people with stroke.

**TABLE 2:** Individuals and groups involved in developing stroke response messaging.

	*n* = 26 respondents who provided information about who was involved in developing their stroke response messaging, %
“If you/your organization uses a different acronym that FAST/BE FAST, who was involved in developing this? Please select all that apply”	
Healthcare professionals	16, 62%
Stroke support organizations	15, 58%
Academics/researchers	13, 50%
Health ministries	5, 19%
Individuals with stroke and/or their family/carers or the public	5, 19%
Other (e.g., Google, public relations specialists and team members)	4, 15%
Who respondents/their organizations collaborated with to develop their stroke response messaging	
1	9, 35%
2–3	15, 58%
4 or more	2, 8%

### 3.5 Dissemination of Stroke Symptom Messages

Among the 56 respondents who completed the survey, all answered the question: “At which level does your organization focus the majority of its awareness activities? (Please select all that apply).” Stroke awareness activities were reported across global (*n* = 50, 89%), local (*n* = 29, 52%), and national (*n* = 5, 9%) levels of organization. Forty‐five respondents (80%) collaborated with other organizations to raise awareness of the signs of stroke, including local organizations (e.g., voluntary or community‐based), regional or national stroke or neurological societies and organizations, governmental health ministries, school boards and global stroke organizations.

Among the 56 respondents who completed the survey, all answered the question: “Who does your/your organization's stroke awareness target? Please select all that apply.” Respondents' stroke symptom messages were aimed at multiple audiences, including the general public (*n* = 53, 95%), younger individuals (*n* = 20, 36%), older individuals (*n* = 22, 39%), health care providers (*n* = 29, 52%) and other groups such as “working people,” “state authorities,” “high risk adults,” “Māori and Pacific communities,” and students (*n* = 11, 20%).

Among the 56 respondents who completed the survey, all answered the question: “How do you/your organization share information about the signs/symptoms of stroke? Please select all that apply.” In terms of dissemination methods, respondents reported using multiple platforms to share their stroke symptom messages. These included television (*n* = 25, 45%), radio (*n* = 26, 46%), print media (*n* = 36, 64%), social media (*n* = 53, 95%), websites (*n* = 46, 82%), public events (*n* = 49, 8%) and other platforms or locations such as grocery stores, community meetings or outreach, public banners, and school presentations (*n* = 13, 23%).

### 3.6 Effectiveness of Stroke Symptom Messages

Among the 56 respondents who completed the survey, all answered the question: “How do you/your organization know if your stroke awareness activity is raising awareness about stroke and changing behavior? Please select all that apply.” Respondents reported the following indicators of effectiveness related to their stroke symptom messaging which demonstrated stroke awareness and/or behavior change related to their messaging: evaluation of impact on hospital presentation (*n* = 22, 39%), pre‐ and post‐testing (*n* = 19, 34%), time to treatment impact (*n* = 17, 30%), number of emergency calls received (*n* = 12, 21%), and other indicators, such as community knowledge, clinical stroke audits and increased number of media talks (*n* = 7, 12.5%). Sixteen respondents (28%) indicated they did not know whether their stroke response activities led to increased stroke symptom awareness and/or behavior change, while two (4%) respondents answered this question, but did not provide any information on outcome indicators (“not yet raising awareness” and “we didn't measure it”).

### 3.7 Lessons Learned About Stroke Symptom Messages

Respondents shared the lessons they had learned about stroke response messaging regarding three main areas: content (category 1), tailoring (category 2), and effective dissemination (category 3). Table 3 provides a summary of these respondents' insights.

**TABLE 3:** Recommendations about stroke response messaging.

Category	Recommendation
Content of stroke symptom messaging	Convey urgency using simple and straightforward language for easy recall and understanding Help individuals easily identify stroke signs and symptoms, including physical and non‐physical symptoms Instruct individuals on how to respond to stroke symptoms, including the appropriate number to call, care facility to attend Offer guidance on how to support someone with stroke
Tailoring of stroke symptom messaging to fit local context	Fit local context, including appropriate emergency numbers and medical facilities for stroke Present message in local language(s) Suit the local culture and beliefs, including demystifying cultural beliefs that may impact responses to stroke Age/group‐appropriate information
Dissemination of stroke symptom messaging	Use multiple dissemination strategies Engage multiple stakeholders, including local community, grassroots organizations, academics, government Use community‐led approaches to share stroke symptom messages with the public Ensure dissemination of stroke symptom messaging is ongoing (i.e., more than one time) Use media and well‐known individuals to disseminate stroke symptom messages broadly Disseminate stroke symptom messages at places with high traffic, including sports games and festivals

#### 3.7.1 Category 1: Required Content of a Stroke Response Message

Respondents highlighted the need for stroke symptom messages to convey the urgency of immediate action and help individuals easily recognize the stroke signs and symptoms, including early, mild and less obvious symptoms. Respondents also highlighted the importance of guiding public response to stroke symptoms, including which emergency number to call and the appropriate medical facility to visit. A respondent from South Africa noted, “In our context, stroke services at hospitals differ and part of educating is recommending‐in government hospitals to go to specific tertiary hospitals with stroke units. Treatment at local hospitals poor and slow…Private hospitals excellent.” Furthermore, the messaging should assist those supporting someone with a stroke. A respondent from Bulgaria recommended clear and simple messages: “information should be presented carefully and under no circumstances should stressful terms and definitions be used. People don't have time, but they would take time for themselves if they understood immediately the benefit and the immediate necessity…simple, straightforward audiovisual narratives on the key issues.”

#### 3.7.2 Category 2: Tailoring Stroke Symptom Messages

Respondents shared the lessons that they had learned about tailoring stroke symptom messages to fit their local contexts. Based on their responses, language translation appeared to be the most used tailoring approach to address linguistic differences. Translation into multiple languages beyond the country's primary language, including local languages, was reported to be necessary to fit local contexts. For instance, a respondent from Pakistan noted a need to “raise awareness about the BE FAST in local languages besides Urdu.” A respondent from North Macedonia stated that stroke response messaging should demonstrate “Cultural Sensitivity: Be aware of cultural and linguistic differences when promoting stroke awareness.” Beyond language, a respondent from Sri Lanka noted that it was important for the stroke symptom messages to “suit the local culture, beliefs etc. and simple to understand.” Some respondents suggested tailoring the stroke response message to specific groups, including “young people, health providers, individuals with stroke risk factors or certain conditions.” When tailoring to younger individuals, “age‐appropriate education materials” were recommended by a respondent from North Macedonia. One respondent from the Philippines reported “cultural beliefs that have to be demystified” to create awareness of stroke signs and symptoms effectively.

#### 3.7.3 Category 3: Effectively Disseminating the Stroke Response Message

Participants highlighted multiple strategies that they used to disseminate stroke symptom messages within their local context. Respondents emphasized that raising stroke response awareness required a coordinated, multi‐level effort involving stakeholders from grassroots and community‐led initiatives to government institutions. Respondents from New Zealand, North Macedonia, India and Sri Lanka specifically cited the need for community‐led and grassroots‐level dissemination: “Use religious leaders, community leaders to take the message to the public at grassroot level.” The involvement of a famous or “a well‐known personality in the diagnosis” was suggested by a respondent from the Czech Republic and North Macedonia. Additionally, a respondent from India highlighted the need for the stroke response messaging to be ongoing rather than one‐time. Respondents suggested different avenues for dissemination, including sports events (Slovakia, New Zealand, and Slovenia), festivals (Slovenia), media (the Czech Republic and Japan), and social media (the Philippines). In addition, respondents from the Philippines and Nepal suggested local brain injury events or days (e.g., World Stroke Day, National Brain Attack Awareness Week) or health professional events (e.g., Nursing Week) as opportunities to share such messages. Respondents from Brazil and Japan suggested disseminating stroke symptom messages in schools. Respondents indicated that “audiovisual narratives,” video campaigns and storytelling were appropriate for conveying the messages.

## 4 Discussion

Fifty‐six respondents from 46 countries provide details on their use of stroke symptom messages and contextual considerations to fit their setting. Nearly all respondents used stroke symptom messages. However, there were diverse approaches to stroke symptom messaging across countries, including using FAST and BEFAST, locally adapted versions of these or other messages created locally.

Consistent public health messaging may improve stroke symptom recognition and timely response (Chen et al. 2021). However, our findings emphasize the challenges of applying common/standard stroke symptom messages across diverse contexts. Respondents in our study highlighted language barriers, cultural nuances, and varied healthcare infrastructure that can hinder the effectiveness of widely used messages, such as FAST and BE FAST. These mnemonics may be suitable in some contexts, particularly those English‐speaking (Chen et al. 2021; Flynn et al. 2014; Hickey et al. 2018; Hogge et al. 2024; Nordanstig et al. 2017; Wolters et al. 2015), but our findings reveal that their implementation in some contexts may be challenging (Zhao et al. 2018). Moreover, countries with multiple emergency numbers or complex healthcare systems require more tailored messaging that instructs individuals on how/where to seek acute care. Our results support prior literature (Banerjee et al. 2022; Hsia et al. 2011; Sese and Guillermo 2023; Zhao et al. 2018; Zhao and Liu 2016), emphasizing the need for context‐sensitive approaches to stroke symptom awareness communication strategies, considering factors such as language, medical infrastructure and local emergency numbers (Chen et al. 2021; Flynn et al. 2014; Hickey et al. 2018; Hogge et al. 2024; Nordanstig et al. 2017; Wolters et al. 2015; Zhao et al. 2018).

Beyond recognizing stroke, behavior change is a fundamental goal of these messages, as rapid response can improve outcomes (Hickey et al. 2018). However, cultural norms (e.g., the tendency to contact a family member before seeking medical assistance) or cultural beliefs (e.g., the efficacy of medical treatment versus other remedies) can influence health‐seeking behaviors (Hsia et al. 2011). Thus, our findings underscore the importance of locally developing or adapting and disseminating stroke symptom messages as they may enhance effectiveness and reach (Hickey et al. 2018).

Survey respondents in our study reported various strategies for local adaptation of stroke symptom messages that aligned with existing research, including translating existing stroke symptom messages, such as the FAST or BE FAST mnemonics, into local languages (Castro et al. 2022; Waweru et al. 2024). However, challenges with public recall of the translated messages and issues with accurate translation were encountered. These challenges speak to broader issues in translating acronyms from English into other languages, as translating acronyms may change their meaning (Kamil 2020; Kim‐Farley 2024; Mohammed 2022). How effective a stroke symptom message is, and needed adaptations can differ based on the context and population (Bray et al. 2010; Rioux et al. 2022; Zhao and Liu 2016). For instance, in some contexts, the original message or acronym may be retained or the order of letters may be rearranged to better fit with the target language's grammatical structure (Bankole 2006). In other contexts, the message or acronym may be replaced entirely when equivalent words do not exist in the local language (Bankole 2006). While acronyms are widely used in English, they may be infrequent/unfamiliar in other languages, and direct translations may not retain the intended meaning, making them unmemorable or meaningless (Kamil 2020; Kim‐Farley 2024; Mohammed 2022). This challenge led to some respondents creating locally crafted stroke symptom messages in collaboration with their community.

The context impacts intervention effectiveness (Moore et al. 2021). The type of local adaptations needed for public health messages can vary by context and may be in the intervention development or implementation phases (de Boer et al. 2025). Systematic approaches to adapting interventions can help align an intervention with the context (Moore et al. 2021). To effectively tailor stroke symptom messages, there is a pressing need for a framework that guides organizations and communities in the adaptation of stroke symptom messages that resonate with local contexts (Sese and Guillermo 2023; Zhao et al. 2018). Existing guidance on implementing interventions in new contexts (de Boer et al. 2025; Moore et al. 2021), including stroke‐specific guidance (CDC 2024; Sese and Guillermo 2023) is available. However, guidance that can be applicable across multiple health systems is needed. Based on these study findings, the guidance should inform processes to adapt stroke symptom messages (e.g., co‐create a new message or adapt an existing message). In addition, our findings resonate with other research, which notes the importance of determining who stroke symptom messages should target and who should be involved in the message development and dissemination. Involving multiple stakeholders may help ensure the message reaches and aligns with the needs/interest of the target audience(s) (Feigin et al. 2024). Finally, based on our study findings, it is important to measure its effectiveness.

Survey respondents shared what is required to locally adapt and disseminate stroke symptom messages to ensure the information reaches the intended audiences. Additionally, respondents identified the importance of targeting multiple audiences with the message, including children who can share the information with their families (Proios et al. 2022). Since different public audiences were being targeted, framing the message effectively to appeal to each intended audience is crucial (Brownson et al. 2018). Involving key stakeholders is vital in creating successful dissemination strategies (Brownson et al. 2018). While mass media has been a common approach for broad reach, it can be expensive (Tunkl et al. 2023), and its effectiveness in prompting behavioral response after stroke sign and symptom recognition may be limited (Lecouturier et al. 2010). Conversely, social media campaigns have emerged as a more cost‐effective approach in low and middle‐income countries (Tunkl et al. 2023).

## 5 Limitations and Strengths

We could not determine the survey response rate due to sampling strategies (i.e., snowball and purposeful) and could not track completion rates (e.g., unsubmitted surveys), which impact the comprehensiveness and reliability of our findings. Reliability may also be impacted by not being able to clarify any unclear/missing survey responses because the responses were anonymous. In addition, no formal validation or reliability testing was performed before data collection to assess the survey's content validity and reliability. Generalizability is impacted because some countries were not represented in the survey, possibly due to language barriers or the organization not being affiliated with the two organizations through which recruitment occurred. In addition, we did not collect respondents' demographic details such as age, gender, and years of experience in the stroke field, which may also reduce generalizability. The study strengths are its widespread reach (i.e., respondents from 46 countries), which may be due to the survey being available in English, Spanish and French languages, reducing some participation barriers.

## 6 Conclusions

Our findings highlight the challenges of applying the same stroke symptom messages across diverse settings. These findings emphasize that stroke messages must prioritize cultural and linguistic adaptability to ensure relevance and effectiveness across diverse populations. Developing stroke symptom awareness tools that are co‐created with local stakeholders such as health care professionals, individuals who have had strokes, and community leaders, and considering factors such as language, medical infrastructure, and local emergency response systems and protocols may enhance message uptake and effectiveness, ultimately improving timely recognition and response to stroke signs and symptoms worldwide. We can improve stroke‐related outcomes globally by equipping communities with the tools to create, disseminate and evaluate stroke symptom messages that are locally relevant and culturally appropriate.

### 6.1 Clinical Resources

Tadi and colleagues (2023) provide a list of acute stroke signs for nursing diagnosis and health teaching and promotion: https://www.ncbi.nlm.nih.gov/books/NBK568693/. The American Stroke Organization provides an overview of stroke symptoms and the FAST stroke symptom message: http://stroke.org/en/about‐stroke/stroke‐symptoms.

## Funding

H.S. holds the Heart and Stroke Foundation of Canada's New Investigator Award.

## Ethics Statement

Ethics approval was granted from the University of Toronto and University of Texas Research Ethics Boards.

## Conflicts of Interest

S.B. and M.L.A.N. are affiliated with the World Stroke Organization.

## Data Availability

Data supporting this research are not publicly available due to ethics restrictions, but may be available upon request.

## Appendix A Survey Questions

1. Which country are you/your organization located in?
2. Please select the category which best applies to you (please select all that apply)
3. Which acronyms do you use to raise public awareness of the signs and symptoms of stroke? (Please select all that apply)
4. If the acronym you/your organization uses to raise public awareness of the signs/symptoms of stroke is different from FAST/BE FAST, have you:
5. If you/your organization use a different acronym than FAST/BE FAST, can you tell us why/how this acronym is appropriate/relevant to your context?
6. If you/your organization use a different acronym than FAST/BE FAST, who was involved in developing this? (Please select all that apply).
7. Please upload what you use to promote stroke signs/symptoms awareness outside of English versions of FAST/BE FAST
8. At which level does your organization focus the majority of its awareness activities? (Please select all that apply)
9. Do you/your organization work in partnership with other organizations to raise awareness of the signs of stroke? If so, please give more details?
10. What is the evidence that raising awareness of signs/symptoms of stroke is needed in your country/region (e.g., low presentation at hospital, lack of awareness of the emergency number)?
11. Who does your/your organization's stroke awareness target:
12. How do you/your organization share information about the signs/symptoms of stroke?
13. How do you/your organization know if your stroke awareness activity is raising awareness about stroke and changing behavior?
14. Please share any lessons/recommendations about stroke symptom awareness that could be shared with other stroke organizations.
